# A comparison of work-related physical activity levels between inpatient and outpatient physical therapists: an observational cohort trial

**DOI:** 10.1186/s13104-016-2119-y

**Published:** 2016-06-16

**Authors:** Wayne Brewer, Raluchukwu Ogbazi, Devan Ohl, Jeffry Daniels, Alexis Ortiz

**Affiliations:** Texas Woman’s University 6124 Institute of Health Sciences-Houston, 7600 Fannin Street, Houston, TX 77030 USA

**Keywords:** Physical activity, Physical therapist, Accelerometer

## Abstract

**Background:**

Physical therapists (PTs) work in a variety of healthcare settings with varied levels of physical activity demands placed on them. The purpose of this study is to compare the physical activity (PA) levels between PTs in inpatient versus outpatient environments for one work week using a cross-sectional design.

**Methods:**

Sixty-one PTs (30 inpatient, 31 outpatient) wore a tri-axial accelerometer and inclinometer for one work-week. The number steps-per-day, PA intensities, energy expenditures and postural positions adopted during the work day were recorded.

**Result:**

Significantly longer amounts of time spent sitting was found for inpatient PTs regardless of the significantly higher number of steps-per-day. Outpatient PTs had a higher number of breaks from sedentary activity with those breaks being longer than the inpatient PTs. The percentage of time spent performing moderate-vigorous PA approached significance implying more time was spent performing these types of activities for outpatient PTs. The energy expenditures between the two groups of PTs were not different.

**Conclusion:**

This study compared the differences in physical activity levels between physical therapists who worked at inpatient versus outpatient environment as little is known about their activity levels. Inpatient physical therapists took more steps per day than outpatient physical therapists but the outpatient physical therapists were less sedentary and took more frequent and longer breaks from sedentary activities. The energy expenditures were similar between both types of therapists and this may be reflective of the gender and bodyweight differences between the groups that equalizes the energy expenditures. The findings of this study suggests that there are differences in the physical activity demands between inpatient and outpatient physical therapists. The results of this study may serve dual purposes: (1) employers may be able to more accurately describe the expected physical activity demands to future employees; (2) individuals tasked with preparing PTs to physically manage their work environment can outline training programs that are diverse based on the specific work environment of PTs.

**Electronic supplementary material:**

The online version of this article (doi:10.1186/s13104-016-2119-y) contains supplementary material, which is available to authorized users.

## Background

Physical therapists (PTs) are members of the health care team who work with patients to prevent, improve or manage physical impairments and dysfunctions that lead to disability [1]. PTs often must possess several physical attributes such as muscular strength and endurance, trunk and extremity flexibility and adequate aerobic capacity in order to provide effective interventions to their patients [2, 3]. The physical demand level of PTs has been labeled as “heavy” by the 1993 Leonard Matheson & Ministry of Labor [4] which is defined as an energy expenditure of 6.4–7.5 metabolic energy equivalents (METS) with occasional, frequent and constant lifting of loads that range from 23 to 45, 11 to 23, and 4.5 to 9 kg, respectively.

The physical demands as defined by the U.S. Department of Labor of occupations that are categorized as “heavy” or greater tend to also be classified as “unskilled” due to the lack of formal academic training required to obtain these positions. An inverse relationship tends to exist between educational level, income and occupational physical demand [5, 6]. Paradoxically, PTs in the United States require a minimum of a bachelor’s degree with approximately 30 % of them possessing an entry-level doctoral degree to obtain licensure to practice [7]. Over 50 % of PTs hold an entry-level or post- professional doctoral degree which places them among the approximate 3 % of individuals in the United States who have a doctoral degree. The median income of PTs in the United States is approximately $85,000 which is a salary that is $15,000 higher than the year 2013 median household income of $65,587 [5–7].

There are numerous published reports that describe the expanding role that PTs have in the areas of physical activity promotion. Intuitively, one may assume that based on this area of practice as well as the documented physical demands required to perform the job that PTs would tend to be physically active during their workday to complete their job tasks [8–10]. PTs work in a variety of different settings that range from acute care hospitals, inpatient and outpatient rehabilitation centers, schools and job sites [11]. Most often the type of setting that PTs work in dictates the physical activity demands that are placed on them due to the types of the patient conditions encountered, their work load, the physical characteristics and layout of their workplace [7, 12–14]. Because of these different work settings and the potential variations in physical demand levels required to effectively manage patients in these settings, an understanding of the physical activity levels that are typically encountered by PTs in these varied settings is necessary. Little is known about the physical activity demands of PTs such as: ambulatory patterns, energy expenditures, requirements to maintain of certain postures and positions needed to perform essential patient care duties. An understanding of the nature of these daily physical activity demands needed to execute patient care tasks may serve to prepare PTs for the rigors of the job, reduce physical fatigue and potentially reduce the risks for injury. In addition, enhanced understanding of the physical activity demands routinely faced by PTs has the capacity to: (1) assist those individuals charged with employing PTs to more accurately describe the physical activity demands of the job based on the workplace setting and (2) guide other healthcare professionals to design appropriate rehabilitation programs for injured PTs that are based on the physical demand level based on the type of setting they work in. The objectives of this study was to compare and describe the physical activity levels of PTs who work in inpatient environments to PTs working in outpatient environments over the course of one work week using a cross-sectional design.

## Methods

The study was an observational cross-sectional design that utilized 31 licensed PTs from three different outpatient facilities and another cohort of 30 PTs working in publicly and privately funded acute care and rehabilitation hospitals between June 2013 and May 2014 in the Houston, TX area. The PTs involved in the study were volunteers who agreed to be in the study after reading and signing an informed consent approved by Texas Woman’s University Institutional Review Board. The inclusion criteria were: (1) a licensed PT; (2) worked at least 40 h per week; (3) performed patient care duties at least 80 % of the workday; (4) worked in either an acute care or outpatient-ambulatory care facility. Subjects were excluded if they did not have at least 6.5 h per day for a 5-day week shift or 8 h for a 4-day a week shift of accelerometry data. For the purposes of this study, the operational definition of an inpatient PT was one who performs 100 % of his or her patient care with individuals who are hospitalized; conversely an outpatient therapist performs 100 % of his or her patient care with patients who are not hospitalized, regardless of the patient case-type (orthopedics, neurological, geriatric, etc.). Sampling was performed by convenience by selecting clinics within the Texas Medical Center in Houston, TX and clinics were students were performing their clinical internships.

Tri-axial accelerometers (GT3XP-BTLE; Actigraph,LLC., FL, USA) were used to measure the physical activity level of the subjects at a frequency of 30 Hz. The inclinometer within these accelerometers was also activated to measure time spent in sitting, standing or recumbent postures. The accelerometers were activated within the Actilife software (v6.0; Actigraph, FL, USA) using each subject’s weight, height, race/ethnicity, sex, date of birth, and hand dominance. The means of the following parameters were the variables of interest for this study: (1) number of steps taken each day, (2) time spent performing sedentary (0–99 counts), light (100–1951 counts), moderate-to-vigorous physical activity (MVPA; ≥1952 counts) each day, 3 total energy expenditure (kcals/day, 4) percentage of time spent in sitting, standing or recumbent postures (%) and 5 daily average and average length of sedentary bouts and breaks (Additional file 1). Descriptive variables such as the mean age, height, weight, BMI and gender frequencies were compared between groups. This specific accelerometer has shown to be one of the devices with the lowest variance showing strong associations between activity counts, measurement of energy expenditure, and good responsiveness to different intensities of physical activity indicating strong validity and overall reliability [15, 16]. A sedentary bout was defined as periods greater than 10 min with less than 99 counts. Sedentary breaks were defined at times where sedentary bouts where interrupted by activity (≥99 counts). The daily average of sedentary bouts was the average number of seconds spent performing sedentary activities on a daily basis while the average length of a sedentary bout was the daily average length of each bout. In a similar manner, the daily average of sedentary breaks was the average length of the interruption of sedentary bouts per day while the average length of sedentary breaks was the average length of each break. The subjects were instructed to wear the accelerometer on the right hip during their working hours for one work week. The PTs in this study worked in different settings and thus the number of hours worked each day varied; to accommodate for this, one work week was defined as 32–40 h a week of direct patient care duties which was the number used to obtain the weekly mean values of the aforementioned outcome measures. Each subject was instructed to remove the accelerometer at the end of each work day and during their lunch break. A valid day was defined with a minimum accelerometer wear time of 6.5 h per day for a 5-day week shift or 8 h for a 4-day a week shift. Instances where the accelerometer was worn for periods that exceeded their work day or during rest breaks, this data was removed from the analysis such that only physical activities that relate to their occupational demands were captured. The therapists in this study self-reported any instances when they wore the accelerometer during non-occupationally related tasks.

The percentage of time spent performing sedentary, light and MVPA per day were calculated with the Freedson 1998 algorithms [17]. The means and standard deviations for each physical activity parameter previously described and descriptive variables such as the mean age, height, weight, BMI and gender frequencies were calculated and compared between groups with two tailed, independent t-tests. Gender composition between the two groups was analyzed via Chi square. All data analyses were conducted using a level of significance set at p ≤ 0.05.

## Results

**Fig. 1:**
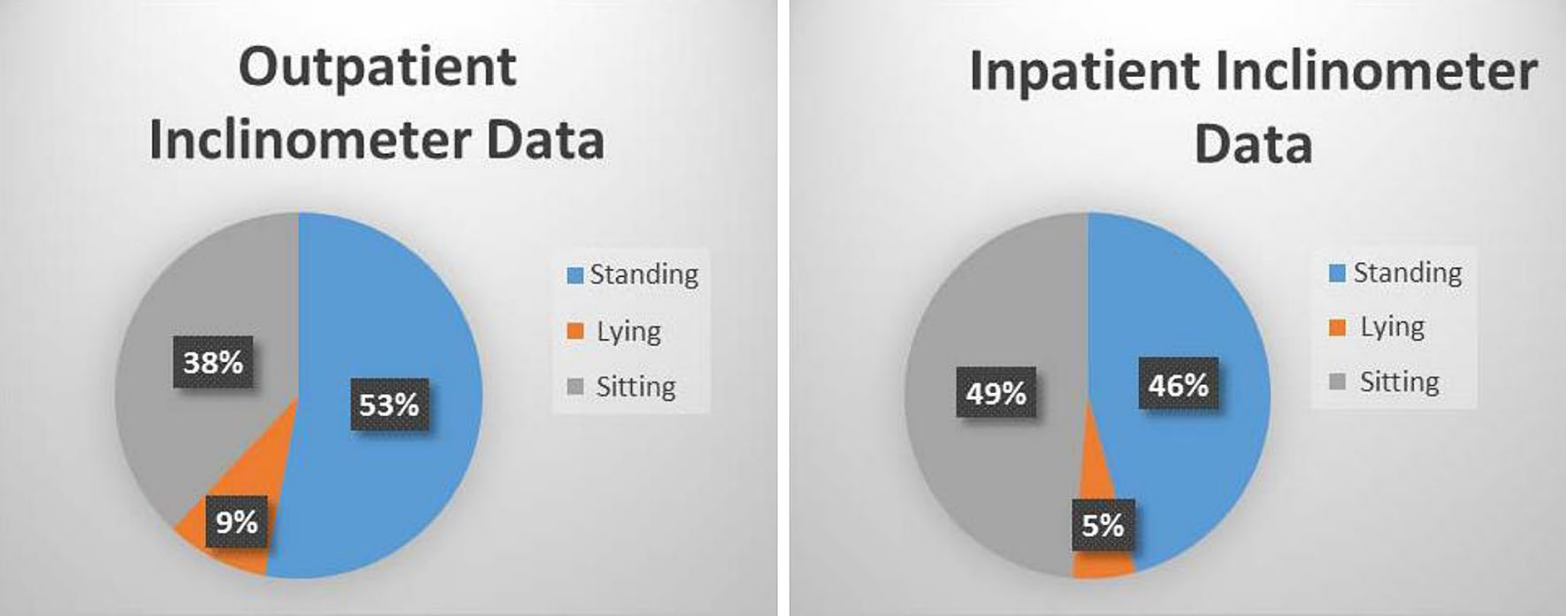
Percentage of work day spent in standing, lying, and sitting

Sixty-one subjects participated in this study; thirty inpatient PTs and thirty-one outpatient PTs. All of these subjects had complete accelerometer data. The outpatient PTs in this study had a significantly higher number of males than females within their group (11 females, 20 males) and between the inpatient PT group (27 females, 3 males). The outpatient PT group had a significantly higher body-mass than the PTs in the inpatient group. Means and standard deviations for all variables for both inpatient and outpatient PTs are presented in Table 1. A significantly higher number of steps taken per day and percentage of time spent performing light physical activities were found for inpatient PTs as compared to outpatient PTs. The percentage of time spent performing moderate-vigorous physical activities approached significance (p = 0.067) implying more time was spent performing these types of activities for outpatient physical therapists as compared to inpatient PTs. Inclinometer data comparing the time spent sitting, standing and lying between the inpatient and outpatient PTs are presented in Table 1 and Fig. 1. Significantly longer amounts of time spent sitting was found for inpatient PTs.

**Table 1:** Physical activity comparison between inpatient and outpatient physical therapists

Variable	Setting	Mean [SD]	Mean difference [95 % Confidence Interval] p value
Age (years)	Inpatient	32.30 [9.01]	1.66 [−2.17, 5.48]
	Outpatient	30.65 [5.55]	NS
Bodyweight (kg)	Inpatient	67.27 [14.06]	−10.66 [−17.96, −3.36]
	Outpatient	77.93 [14.42]	**
Height (cm)	Inpatient	64.43 [2.42]	−3.03 [−4.65, −1.42]
	Outpatient	67.47 [3.70]	***
BMI (kg/m^2^)	Inpatient	25.14 [5.67]	−1.07 [−3.54, 1.40]
	Outpatient	26.21 [3.65]	*
Gender	Inpatient	(90 % female, 10 % male)	***
	Outpatient	(35.5 % female, 64.5 % male)	
Steps-per-day	Inpatient	4475.17 [1464.71]	1280.16 [563.08, 1997.28]
	Outpatient	3195.01 [1333.10]	***
Average kcals-per-day	Inpatient	131.71 [97.58]	−22.60 [−73.55, 28.35]
	Outpatient	154.31 [101.17]	NS
Percentage of sedentary time spent per day	Inpatient	73.79 [7.39]	4.60 [0.40, 8.79]
	Outpatient	69.19 [8.89]	*
Percentage of light time spent per day	Inpatient	20.54 [6.07]	−2.57 [−5.99, 0.84]
	Outpatient	23.11 [7.19]	NS
Percentage of moderate-vigorous time spent per day	Inpatient	5.67 [2.44]	−2.02 [−4.20, 0.15]
	Outpatient	7.70 [5.44]	NS
Percentage of time spent sitting per day	Inpatient	48.56 [10.28]	9.7 [3.57, 16.01]
	Outpatient	38.76 [13.26]	***
Percentage of time spent standing per day	Inpatient	46.05 [11.70]	−7.79 [−14.91, −0.67]
	Outpatient	53.84 [15.25]	*
Percentage of time spent lying per day	Inpatient	5.39 [6.68]	−2.00 [−7.39, 3.38]
	Outpatient	7.39 [13]	NS
Daily Average of Sedentary Bouts	Inpatient	151.99 [165.80]	50.06 [−29.43, 129.56]
	Outpatient	101.93 [144.05]	NS
Average Length of Sedentary Bouts (sec)	Inpatient	30.57 [73.62]	11.21 [−16.42, 38.84]
	Outpatient	19.36 [21.85]	NS
Daily Average of Sedentary Breaks (sec)	Inpatient	1277.24 [439.49]	−453.42 [−746.86, −159.97]
	Outpatient	1730.65 [676.85]	***
Average length of Sedentary Breaks (sec)	Inpatient	239.08 [170.89]	−202.72 [316.38, −89.06]
	Outpatient	441.80 [261.76]	***

* p < 0.05, ** p < 0.01, *** *p* < 0.001

## Discussion

This study is one of the first published reports that quantitatively describe the physical exertion levels encountered by physical therapists working in different settings. In general, the physical activity levels of a PT are not sufficient to promote improvements in health and fitness as suggested by the guidelines set forth by the Federal government [18]. Despite the physical demand level required of a physical therapist as being “heavy”, this may be reflective of the muscular strength efforts required to be a physical therapist and not the cardiovascular demands placed on them. Previous studies have shown that PTs tend to be keenly aware of the need for a structured physical exercise program to promote optimal health for their patients and themselves [19–22].

The fact that inpatient PTs took approximately 30 % more steps during their workday than the outpatient PTs was expected. Typically, hospital campuses have larger square footage with multiple departments and units that a PT must access for patients that require treatments at their bedside or need to be retrieved at their bedside and taken to the rehabilitation department for their care. Outpatient rehabilitation centers typically work with patients who are either ambulatory or patient who are brought to the center by another individual using assistive devices. The nature of the work performed by outpatient rehabilitation therapist may limit the requirements for therapists to walk during their workday. When compared to other healthcare professionals working in the inpatient environment, our study reported lower step counts for PTs as compared to the physicians working in an urban medical center [23]. They assessed the average daily step counts for general cardiologists, CT surgeons, procedural cardiologists, and cardiac anesthesiologists using a pedometer and found daily step counts of 6540, 6039, 5910 and 5553, respectively. It is important to note that the physicians in his study worked an average of 12.3 h a day with the exception of the cardiac anesthesiologists who worked an average of 9.3 h per day which are comparable work-hours to most of the PTs in our study who averaged 7.32 work hours per day. The step counts of the outpatient and inpatient PTs in the present study had considerably lower daily step counts than the cardiac anesthesiologists in the aforementioned study (4475 and 3195 vs. 5553 steps per day). The step counts displayed by these physicians are similar to the 7333 average steps per day taken by emergency room residents working in an urban hospital setting as described in another published study [24]. It is important to note that the PTs in our study worked in medical centers located in urban areas which tend to be more crowded, have higher census levels and larger number of staff as compared to smaller, more rural medical centers.

Reports such as this present study and those described previously sought to delineate the occupational physical activity levels of healthcare practitioners from observational designs. Inferences from these studies, however must consider several potential confounding factors. Patient census levels that vary over time may affect the levels of occupational physical activity displayed by clinicians. None of these reports described the square footage, proximity of their facilities and departments to each other that. Misinterpretation may occur in studies that report the occupational physical activity levels of clinicians who work in spacious facilities but perform the majority of their patient procedures at wards that are proximal to departments that they are located in, which would minimize the need to ambulate to encounter patients. The type of environmental setting, such as urban versus rural is frequently described in published reports, however that description may need to be coupled with the number beds, square footage of the facility and the campus it is located on. Accounting for these factors may allow for more accurate comparisons to be made across studies of different practitioners at varied types of healthcare facilities. In addition, most of the physical therapy care delivered in the inpatient setting is typically done during daytime hours. These hours are times when operations at a hospital are high and thus may pose a barrier to ambulate during the day due to crowding, equipment use, productivity requirement and the availability of hospital escorts to retrieve patients. To date, there were no published reports that examine the step count or energy expenditures of healthcare professionals who work in an outpatient setting on a full time basis but we speculate the similar factors may affect the number of steps taken per day for these therapists as well.

An interesting finding is this study was the fact that despite having a higher step count, the inpatient PTs in this study spent a larger percentage of their time performing sedentary physical activities. The accelerometer that was used measures steps taken per day similar to a pedometer, however the step rate, expressed as steps per minute and alterations in bodily acceleration [counts] are used in combination with the inclinometer data in an algorithm [25] to classify whether an individual is engage in sedentary, light or MVPA or not [17]. Some plausible explanations may be the inpatient therapists walked more at a leisurely pace as compared to outpatient physical therapists during their work day and typically, the caseloads for inpatient PTs are more conducive for one to one treatment encounters whereas outpatient PTs often are in clinics where they must treat multiple patients at a time. We hypothesize that this variance in work flow may be more conducive to a faster walking pace to meet the outpatient physical therapists’ physical activity demands.

It is important to note that despite the fact that the outpatient PTs in our study displayed less time spent performing sedentary activities, the average energy expenditures expressed as kcals-per-day were not different between the two groups. There are several plausible explanations for this finding. Despite the outpatient PTs in this study being less sedentary, they did not engage in activities that were intense enough to increase energy expenditure. Although previous studies were able to extrapolate energy expenditures from ambulatory activities in healthcare professionals, it is important to note that the energy expenditures from PTs may not be captured fully by the use of accelerometer [23, 24]. Physical therapists typically engage in diverse types of physical activities during their workday. Accelerometry only records bodily movements that create acceleration in one or more of the cardinal planes of motion. Many of the physical tasks performed by PTs working in either inpatient or outpatient environments utilize bursts of high intensity activities such as transferring a dependent patient from one surface to another, lifting or performing isometric movements that require a sustained muscular contraction such as holding or supporting a patient in an upright position or performing a manual mobilization technique to a joint. Accelerometry coupled with additional instruments to measure heart rate, bodily temperature and perspiration rates may be necessary to capture the in vivo energy expenditures related to the unique but common tasks performed by a physical therapist. One possible reason for the similarities in energy expenditures may be due to the disparities found in gender and bodyweight between the two groups. The outpatient PTs in our study were significantly heavier and had a higher proportion of males than the inpatient counterparts; these differences may have allowed them to expend similar amounts of calories during their workday with less physical activity. The accelerometer software determines energy expenditures based on an algorithm that includes as factors, bodyweight and gender among others; which would support the previous notion of individuals with higher bodyweight may expend similar amounts of energy with less physical activity than their light weight counterparts [17]. Another possible reason for this disparity is the inherent inaccuracy of this accelerometer to estimate energy expenditure. It has been reported that the Actigraph accelerometer tends to overestimate low levels of activity and overestimate more vigorous activities. However, the Freedson equation used in our analysis has the closest reported correlation (r = 0.33; p < 0.05) to indirect calorimetry for typical walking activities. Therefore, it is assumed that the estimation of energy expenditure used in this study is the closest estimation possible to the therapists’ energy expenditures [26].

Even though the inpatient PTs exhibited an 11 % greater time spent sitting than the outpatient PTs, the inpatient and outpatient therapists in this study sat for approximately 49 and 38 % of their workday and stood for 53 and 46 % of their workday, respectively. These percentages equate to upwards of 4 h of sitting and 4–5 h of standing per day for both positions. The bodily positions adopted during the participants’ workday were measured by the inclinometer, which accounts for position only and not that is energy expended while in those positions. It is conceivable that a PT could exhibit higher energy expenditures while sitting if they are involved in lifting, supporting or positioning tasks which are often required to implement a patient intervention. Conversely, it is possible that the adoption of the standing position does not necessarily infer higher energy expenditures beyond sedentary levels, particularly if the individual is standing still or leaning against a treatment table or wall while standing. These phenomena may explain the paradoxical findings of more frequent standing and less frequent sitting displayed by the outpatient therapists as compared to the inpatient therapists in this study. Both groups of therapists spent similar amount of time in sedentary bouts and those bouts were of similar length. However, when we observe the breaks in sedentary time, outpatient therapists significantly broke sedentary bouts more times during their workday than inpatient therapists and those breaks were twice as long as the inpatient therapists. The amount of these breaks and their length are the main factors that account for the difference spent in sedentary behavior and standing activities between the groups of therapists. Previous published studies have demonstrated that significant reductions occur in metabolic and muscular activity after prolonged sitting [27–29]. These decrements in metabolic and muscular activity have been attributed to the progression of deleterious metabolic derangements such as hyperglycemia, dyslipidemia and hypertension [30]. Some of the adverse effects of prolonged sitting can be ameliorated with regular, brief movement breaks such as standing and walking [27]. The accelerometer data revealed that both groups of therapists interrupted their sedentary bouts frequently throughout their workday which is a behavior that appears to be attributable to the physical activity demands of the job and thus these behaviors may have a protective effect on reducing the incidence of diseases attributed to prolonged bouts of occupationally induced sedentarism.

The implications of this study may allow for a more refined description of the physical activity demands of the work of PTs. PTs work in varied patient care environments. Employers, State and Federal occupational agencies charged with ensuring a safe and injury-free workplace need to consider the possibility of disparate physical activity demands required to perform the job safely. Numerous studies of occupationally related injuries of PTs have found an increased risk of musculoskeletal injuries with increased physical fatigue [2, 3, 13, 14, 31]. Formal and informal educational programs tend to focus on biomechanical principles of lifting and transferring of patients; little attention is paid towards the enhancement of aerobic fitness, musculoskeletal endurance, flexibility and strength as additional possible methods to reduce occupational injuries. This study outlined the additional physical activity demands of PTs that were in addition to the lifting requirements described by occupational agencies such as the US Department of Labor. This further delineation of physical activity demands may allow for more specifically designed rehabilitation programs for injured PTs that are tailored based on the distinct characteristics of their healthcare setting.

The generalizability of our findings is a limitation of this study. The therapists who worked in the inpatient settings were predominately female and the opposite was true for therapists in the outpatient setting. Previous studies have documented gender differences in energy expenditures and physical activity levels whereas this study did not analyze the data across gender [32–34]. Despite the physical therapy profession being comprised of approximately 70 % women, this disparity is minimized in outpatient settings [7]. The reasons for the absence of a separate analysis by gender in our judgement improves the generalizability of our findings to mirror the proportions of other inpatient and outpatient settings that have similar percentages of male and female therapists working in them. Future studies that seek to compare physical activity levels and energy expenditures between PTs that work in different settings may need to consider quota sampling to ensure a more equal distribution of genders for analysis.

The inferences from this study would have been strengthened with the inclusion of interviews for the PTs who wore the accelerometer to attempt to elucidate their daily work conditions, number of patients treated and perceptions of physical effort experienced during this time period. Use of these interviews may serve to confirm or refute the speculative explanations for the larger percentage of the workday spent performing sedentary activities for the inpatient PTs despite their higher step counts as discussed previously. Semi-structured interviews can clarify how various postures such as sitting or standing were adopted. Having access to this information may allow for distinctions to be made among different levels of standing or sitting, particularly if external support is involved (i.e. leaning on an object while standing). The use of these interviews can allow future researchers to account for the use of static postures and positions that are adopted when activities such as lifting, holding, pushing or pulling are performed to improve the accuracy of the energy expenditure calculations.

In summary, despite the step counts being lower for PTs in both types of settings than other health-care professionals, future studies that aim to describe activity levels of healthcare professionals may need to factor the hours worked per day, the shift type (daytime vs. nighttime) and the structural and environmental factors of the facilities they work in. Lastly, the physical activity demands of most PTs are episodic in nature; utilization of multiple modes to assess energy expenditure such heart rate, gait speed, heat flux and perspiration may be necessary to variety of muscle contractions performed by PTs. This study compared the differences in physical activity levels between physical therapists who worked at inpatient vs. outpatient environment as little is known about their activity levels. Inpatient physical therapists took more steps per day than outpatient physical therapists but the outpatient physical therapists were less sedentary and took more frequent and longer breaks from sedentary activities. The energy expenditures were similar between both types of therapists and this may be reflective of the gender and bodyweight differences between the groups that equalizes the energy expenditures. The implications of these findings to the study of occupationally based physical activity are energy expenditures has demonstrable relationships with the prevention of multiple diseases. Description of occupational based physical activity levels should consider factors other than volume (i.e. steps per day). Factors such as gender, body mass, intensity of the work tasks, frequency and number of work breaks taken needs to be elucidated. Physical activity assessments must include the intensity of the work tasks performed.

## Additional file

10.1186/s13104-016-2119-y Accelerometry data.

## Abbreviations

PTs: physical therapists
METS: metabolic energy equivalents
MVPA: moderate-to-vigorous physical activity
kcals/day: total energy expenditure
